# Method for evaluating roughness and valley areas coefficients of surfaces acquired by laser scanner

**DOI:** 10.1038/s41598-022-04847-2

**Published:** 2022-01-27

**Authors:** Leandro Tonietto, Daiana Cristina Metz Arnold, Valéria Costa de Oliveira, Camila Werner Menegotto, Atilio Efrain Bica Grondona, Cristiano André da Costa, Mauricio Roberto Veronez, Claudio de Souza Kazmierczak, Luiz Gonzaga

**Affiliations:** 1 Graduate Program in Applied Computing, Unisinos University, São Leopoldo, RS Brazil; 2 VIZLab Advanced Visualization & Geoinformatics Lab, São Leopoldo, RS Brazil; 3 Feevale University, Novo Hamburgo, RS Brazil; 4 Graduate Program in Civil Engineering, Unisinos University, São Leopoldo, RS Brazil; 5 Federal Institute of Rondônia, Porto Velho, RO Brazil; 6 SOFTWARELAB (Software Innovation Laboratory), Unisinos University, São Leopoldo, RS Brazil

**Keywords:** Computer science, Civil engineering

## Abstract

The quantitative determination of average roughness parameters, from the determination of height variations of the surface points, is frequently used to estimate the adhesion between an adhesive and the surface of a substrate. However, to determine the interaction between an adhesive and a surface of a heterogeneous material, such as a red ceramic, it is essential to define other roughness parameters. This work proposes a method for determining the roughness of red ceramic blocks from a three-dimensional evaluation, with the objective of estimating the contact area that the ceramic substrate can provide for a cementitious matrix. The study determines the average surface roughness from multiple planes and proposes the adoption of 2 more roughness parameters, the valley area index and the average valley area. The results demonstrate that there are advantages in using the proposed multiple plane method for roughness computation and that the valley area parameters are efficient to estimate the extent of adhesion between the materials involved.

## Introduction

Pathological manifestations in mortar coatings applied on ceramic block substrates are frequent in civil construction, and are often caused by the lack of standardization of the coating system^1^. The adhesion between the cementitious matrix and the substrate results from the union between the tensile adhesion strength, shear adhesion strength and the adhesion area, defined by the ratio between the effective contact area and the total area possible to be joined, being these, properties of the contact region between the two materials^2,3^. The main reason for the fall of coatings is the poor adhesion between the mortar and the substrate, mainly caused by the lack of information about the characteristics of the substrates and the inadequacy of the rheology of the coatings^4–6^.

The roughness of the substrates can provide a greater adhesion area between the substrate and the coating, but the rheological properties of the mortar related to the contact area and substrate roughness determine a greater or lesser adhesion between them. Adhesion between ceramic blocks and coating mortars is also conditioned by the absorption of hydration products from the cement paste by the pores of the substrate, which when hydrated promotes a mechanical anchorage of the mortar to the substrate.

The geometric characteristics of the surface, at the macroscopic level, influence the adhesion area. Vaz^6^ identified that the bond strength with the presence of roughcast is around $35\%$ higher than on the smooth surface and when the blocks have grooved surfaces they present an average increase of $12\%$. The influence of the roughness of ceramic blocks on the adhesion of coatings needs to be discussed, especially when it comes to surfaces that macroscopically are considered smooth.

The mechanical anchorage model proposed by^7^ deals with the penetration of the cementitious matrix in the valleys and pores of the substrate as the main factor in determining the adhesion. What is also pointed out in^8^, which mentions that the surface roughness of materials directly influences the adhesion of coatings, since the tendency is that by increasing the surface roughness, the contact area is also increased. In addition to mechanical anchoring, adhesion forces on the surface of materials, given by Van der Waals forces, polar covalent bonds between particles at the interface and chemical adhesion^9^, can contribute to adhesion in a small proportion.

Related to mechanical anchorage adherence, Polito et al.^10^ mention that the hydration products of the mortar paste binders penetrate to depths between 100 and $1600~\upmu m$ inside the substrate and that the rupture region where adhesion loss occurs in a layer adjacent to the substrate/mortar interface with approximately 50 to $200~\upmu m$. However, the studies carried out cannot be generalized yet, mainly due to the lack of a recognized method to show the effect of roughness in increasing the contact area of the cement matrix with the substrate. In this context, the interfacial adhesion strength of the coatings depends on the morphological characteristics of the interface region^11^ and the contact area between surfaces, which is dependent on the substrate roughness and the characteristics of the cementitious matrix of the coating.

In Scrivener et al.^12^ suggest that adhesion depends on the geometric compatibility of the minimum particle size of the cementitious matrix and the area of roughness formed by peaks and valleys, in order to reduce contact failures between the materials. Thus, the aim is to develop a model that shows the mechanical interlocking of the cementitious matrix composites of coatings in the valleys and pores of the red ceramic blocks.

In the case of ceramic block surfaces that, on a macro-scale, are considered smooth, the proper adhesion of a surface is even more complex than the simple measurement of peaks and valleys. Measuring surface roughness requires the use of different mathematical algorithms, which can lead to different results from the same data entries^13^. However, in^14^ the author comments that the adhesion of the mortar on the substrates can be harmed by rougher surfaces. Another work built on this approach is^15^, the authors used two different surfaces, one smooth and the other striated, justifying that the rougher surface showed increased adhesion. Ghumatkar et al.^16^ verified that there is an ideal surface roughness for maximum adhesion and the roughness range depends on the adhering material. Thus, there are different results of roughness analysis depending on the scale of the study carried out, since the perception that the adequate parameterization of a surface is even more complex than the simple measurement of peaks and valleys.

Most works where roughness is quantitatively calculated use two-dimensional evaluation methods. However, the two-dimensional analysis has been criticized and new solutions are proposed to describe the surface roughness^17–19^, since a single profile does not adequately characterize a 3D surface, as the focus on the surface macrotexture is always limited because the surface is qualitatively classified, and the average roughness $R_a$ is not sensitive enough, as it does not provide information about the local variability, as different profiles can have the same $R_a$. What is stated in the study by Arnold et al.^20^, which compared the roughness of ceramic substrates using 2D and 3D methods, the results emphasize that in the 2D method, the measurement orientation strongly influences the result and that the 3D method allows to identify variations in roughness typical of a ceramic block.

This work proposes a 3D measurement method that makes it possible to characterize the roughness of red ceramic blocks with emphasis on the contact area of adhesion between the substrate and the cementitious matrix.

## Related works

For substrate roughness analysis there are several studies^21–27^, which adopt different quantification methods, and there is no indication on the ideal method for defining roughness for ceramic blocks^26^. In this sense, Perez et al.^24^ indicate the use of more than one method to obtain the real roughness of the surfaces in general.

Roughness parameters are numerical quantities, based on geometric characteristics of spacing, height and depth between peaks and valleys and are usually obtained using 2D profiles or 3D surfaces^26^. Among the most used parameters for surface roughness quantification is the average roughness ($R_a$), which is the arithmetic mean between the peaks and valleys of the surface and the $R_q$ which is the root mean square of the $R_a$^26,28^.

However, studies such as^23,29^ point out that the determination of $R_a$ and $R_q$ are inefficient parameters to represent the visualization and quantification of the surface roughness analyzed, since different profiles can be obtained of roughness with different characteristics, but with the same value of $R_a$. Other works^30,31^ state that the $R_a$ and $R_q$ represent a profile of a statistical mean of the surface and not the real roughness of the analyzed substrate.

As already shown by^20,21,32,33^ the $R_a$ has significantly different values depending on the measurement location. This difference is clearer when the surfaces analyzed are composed of heterogeneous materials such as red ceramic substrates.

It is also important to understand the scale of application of the method, since the literature points out different scales of surface topography for the study of roughness, even obtaining different levels of roughness according to the scale expansion^23,26,29^. Roughness is treated from the level of waviness or macroroughness (between 0.5 mm and 50 mm), but it is from the micrometer scale that it is called roughness itself (between $1 ~\upmu m$ and 0.5 mm)^20,34^.

The three-dimensional methods for data acquisition and roughness calculation are the most indicated^20,21,23^, as they present more accurate results in relation to the microscale, as they consider sampling of the entire surface area regardless of reading position or direction and also because, in general, they use equipment with better resolution and reading accuracy^20^. The most used equipment for 3D measurement is based on a laser scanner (eg LiDAR), which emits a beam of light and records information about its return^20,21^, forming a 3D point cloud of the surface.

Acquisition of the point cloud with surface data is an important step that requires attention to the method of data acquisition and reading. Care must be taken to avoid failures associated with the production and data acquisition steps, to prevent interference from particles or elements that bring imperfections. The authors^20,21,35^ describe the different types of problems, and propose care in handling the material and calibration of the reading equipment that favor the quality and quantity of the data acquired^21^.

Tonietto et al.^21^ define a method to quantify the roughness of red clay ceramic blocks based on data obtained by a 3D scanner. The method enables the localized evaluation of roughness parameters, enabling the effective determination in the control and comparison of roughness of red ceramic substrates produced with heterogeneous materials. The authors describe various procedures from data acquisition, calculation of fitting plane, roughness calculation and the representation of information in levels of detail to roughness evaluation forms. The method that performs the calculation process for surface roughness from a single fitting plane proved to be effective in determining the average roughness $R_a$ in the context of smoother surfaces, such as red clay ceramic substrates, where the samples they do not have much variation in waviness (macroroughness). However, in cases where the surfaces have greater waviness or where it is intended to analyze with a greater level of detail, the method has limitations, mainly because it only considers a single fitting plane for the entire surface.

The fitting plane is computed by calculating the least squares product, which establishes a regression of the point cloud to an fitting or smooth surface^21^. To perform the calculation of the product of least squares, in^21^, the matrix form is used. The information in *x* and *y* of each point composes the geometry matrix (*A*) of the surface. The *z* of each point is used to compose the height matrix (*L*).

$$\begin{aligned} A = \begin{bmatrix} 1 &{} x_0 &{} y_0 \\ 1 &{} x_1 &{} y_1 \\ \vdots &{} \vdots &{} \vdots \\ 1 &{} x_n &{} y_n \end{bmatrix} \qquad L = \begin{bmatrix} z_0 \\ z_1 \\ \vdots \\ z_n \end{bmatrix} \end{aligned}$$

From the multiplication of the matrices, the coefficients that define the fitting plane (*B* matrix) are found. The equation used to perform the least squares product operation is adapted to adjust the dimension of the matrices to make the multiplication between them compatible:

$$\begin{aligned} B = (A^T \times A)^{-1} \times (A^T \times L) \qquad B = \begin{bmatrix} b_0&b_1&b_2 \end{bmatrix} \end{aligned}$$

From the coefficient matrix (*B*) it is possible to compute the height of a point *p* in relation to the fitting plane of the sample. In this way, the roughness at a given point is computed based on the distance from the point’s *z* to the plane’s *Z* at the same coordinate *x*, *y*:

$$\begin{aligned} Z = b_0 + b_1 \times x + b_2 \times y \end{aligned}$$

The average roughness ($R_a$) is calculated after the hierarchical subdivision of the structure and for each region (node) of each level of the structure, the roughness parameter ($R_a$) is calculated similarly to the formulation described in^26^:

$$\begin{aligned} R_a \approx \displaystyle \frac{1}{mn} \sum _{j=1}^{m}\sum _{i=1}^{n} |z_{ij}| \end{aligned}$$

where $Z_{ij}$ is the distance from *z* of ponit ($p_{ij}$) to *z* of plane *P*. After the calculated values of $R_a$ and the other parameters already mentioned and suggested in^26^, the method^21^ defines a roughness signature to represent the roughness in different regions on the surface and tools for visual and quantitative assessment of roughness. Allowing to verify the behavior and distribution of roughness along the surface.

Although the reference works^5,23,26,36^ use the average roughness parameter ($R_a$) as a roughness analysis criterion, it may fail as the only measure of adhesion evaluation by area of contact, as it is a measure related solely to height and no horizontal or area measures are considered in the evaluation. In addition to roughness, several authors agree^5,6,37^ that other factors also impact the bond strength, such as porosity, water absorption and contact area^37^.

Regarding the contact area, for example, in cases where the area of a valley on the surface of the substrate is less than the depth of the region, a spherical cement particle that has a diameter greater than the valley area would not be able to penetrate this it is valid, even if the depth ($R_a$) is greater than its diameter. In this sense, Kozubal et al.^34^ suggest the concept of density of particles that tend to come into contact with the surface as a way to determine the bond strength between concretes of different ages. Pour^8^ also states that the $R_a$ cannot be used as a single measure to determine adhesion, as it is an average of the entire surface.

In addition to quantifying the peaks and valleys of a surface, the surface area will directly influence the mechanical interlock between the coating and the substrate, promoting greater or lesser surface adhesion. It is estimated that the greater the penetration of particles of the cementitious matrix in the surface valleys, the greater the proximity to the substrate and, consequently, the greater the adhesion area. Saho et al.^38^ also state that the surface texture of the substrate can be influenced by the production process (such as grooves caused by the equipment) and also that surface imperfections interfere in the adhesion area^39^.

Regarding the adhesion between ceramic substrates and coatings, considering the contact area, the geometric information of this work allows the analysis of areas or regions where there is favoring of a higher concentration of particles of the cement matrix in the region of interface with the substrate and, consequently, greater adhesion area.

## Method for surface roughness and valleys area coefficients evalution from multiple planes

**Figure 1:**
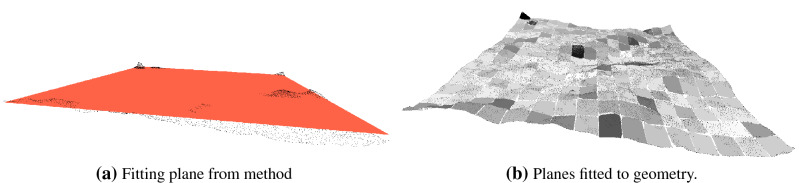
Planes calculated on the same point cloud. In (**a**) the fitting plan calculated with the method proposed by Tonietto et al.^21^. In (**b**) the planes are fitted to the sample geometry.

In this work, an modification in the method described in^21^ is proposed to consider the local variations of the geometry and also to change the way of calculating the distance between the point and the plane. The method described in^21^ only calculates a single fitting plane for the point cloud (see Fig. 1) and the roughness is calculated through the absolute distance of each point *z* to the plane *z*. Therefore, for all regions or nodes of the spatial subdivision, the same plane is used to compute the roughness coefficients. The local variation of the points is not considered and the roughness values that are computed can be overestimated, precisely because they do not consider the local geometry of the points.

On surfaces with a higher level of waviness, the single fitting plane considers these waviness (reliefs) as part of the roughness, interfering with or accentuating the roughness values for the surface. Figure 1 shows the difference between a average plane calculated with the method described^21^ and an example with several planes calculated taking into account the local average surface, from the same point cloud.

### Using principal component analysis for planes computation

The first modification needed to account for local variations in geometry is the computation of local fitting plane for each surface region. In this way, the roughness parameters associated with a region or surface location are calculated based on the local plane and no longer in relation to the global plane.

The modification in relation to the method described in^21^ takes place in two important points: first, the change in the order of the algorithm for calculating the plane and the division of areas; second, using the principal component analysis (PCA) method^40^ to calculate the plane associated with the points at each location.

**Figure 2:**
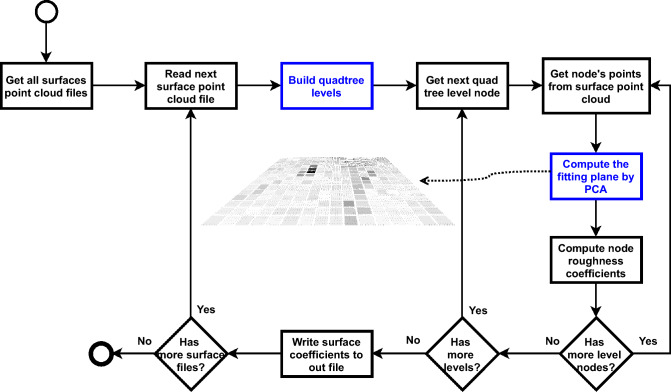
Process for calculating the fitting plane for each surface location, at all levels of hierarchical division.

The flow of the fitting plane calculation process is shown in Fig. 2. The steps that are highlighted are the main modifications of the general process of the method described in^21^. The construction of the quadtree is performed first and the plane calculation is performed for each node at each level of the hierarchical structure.

In the method presented in^21^ the fitting plane is computed considering the axis *Z* as height and the roughness is determined by the absolute difference of the value of *z* of each point in relation to the *z* of the plane, in same coordinate *XY* ($a = P_{[i]}.z - getZ(plane, P_{[i]}.xy$)). Consequently, distances from the plane are always orthogonal to *XY* plane.

When data is acquired from a flat and leveled surface, the orientation angle of a single plane is close to zero and the difference in *z* to define the height is not a problem. However, if the local planes are considered, there is a significant variation in angle, and it is necessary to calculate the distance of the point in relation to the plane independently of the orientation of this plane.

**Figure 3:**
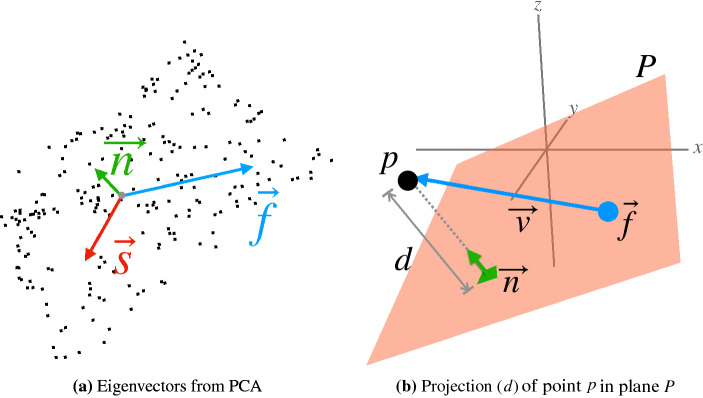
PCA components computed for a set of points (**a**) and in (**b**) point-to-plane distance calculation.

For computing multiple local planes, the method proposed in^40^ is used, which analyzes a point cloud and calculates three eigenvectors (and respective eigenvalues) that define each plane. The first eigenvector represents the largest dispersion of the data, the second is orthogonal to the first in the direction of the second largest dispersion of the values and the third is perpendicular to the two previous ones and represents the third largest dispersion of the data and is also the normal vector (or direction) of the plane. Thus, an fitting plane *P* of a set of points *NP* is a system defined by three vectors: $P(NP) = (\vec {f}, \vec {s}, \vec {n})$, where $\vec {f}$ and $\vec {s}$ are the two vectors that define the base of the plane and $\vec {n}$ is the direction of the plane. To calculate planes fitted to the original surface, the process must be performed recursively dividing the point cloud associated with the current plane into two halves. For each half, the same process of computing planes through eigenvectors is performed. The process is repeated until the minimum limit of points per plane or reaching the maximum number of subdivisions. Figure 3a illustrates the representation of eigenvectors ($\vec f$, $\vec s$ and $\vec n$) of a set of points.

For the method proposed in this work, as shown in Fig. 2, the subdivision of the point cloud occurs before the calculation of the planes, so that there is a uniform division of the area of each node or region, following the rule quadtree division (as defined in^21^). The original method^40^ was adapted and defined to not allow subdivisions and the parameter of minimum number of points was also adjusted to 4, in order to have a minimum geometry for calculating the plane. In summary, the PCA (3-vector system) plane calculation method is executed for each node of each level of the quadtree.

After calculating the plane for each node, the average roughness parameter $R_a$ is computed at each node of each surface level. For each point associated with the region/node, the distance from the point to the node plane is calculated (as illustrated in Fig. 3 (b)). Then the roughness parameter calculation procedure follows the calculation formula as defined in^21,26^.

The distance *d* from a point *p* to the plane *P*, is obtained through the dot product of the vector that goes from the plane to the point with the normal of the plane. Therefore, $d = \vec {v} \cdot \vec {n}$, where $\vec {v}$ is the vector that goes from the first eigenvector of the plane system to the current point in the cloud: $\vec { v} = p - \vec {f}$. Therefore, *d* is the projection of the point *p* in the plane normal. Figure 3b demonstrates the parameters involved and the relationship between them.

Finally, the roughness data are made available and analyzed in the same way as described in^21^.

### Valley areas calculation

The method proposed in this work for the analysis of contact areas, classifies surface regions that are valleys and identifies lakes that form in these regions, where there will be a tendency for a higher concentration of binder grains if, on the substrate under analysis, a cement paste. Lake areas are computed to determine the total valley area, or surface where the agglomerates grains may come into contact with the substrate surface.

Two parameters are proposed for the analysis of the valley area: the *valley area index* ($\triangle {_T}$) and the *average valley area* ($\triangle {_{avg}}$). The calculated value for the valley area is the ratio of the sum of the lake areas to the total surface area. This guarantees a normalized value, free from variations in surface dimensions. The parameter $\triangle {_{avg}}$ is the total area of valleys divided by the number of valleys.

For the computation of the lakes that form the valleys, only the regions that have edge points (lake boundary) and at least one internal point (which does not belong to the edge) are considered. The edge is the boundary at the height of the plane of the region (or boundary defined according to the selected level parameter). For example, a point where the height (distance from a point to the plane) is zero is a point that is exactly above the plane. By default, this level parameter is set to zero, hence the height of the local midplane. All points at or below the selected level are considered valley points.

**Figure 4:**
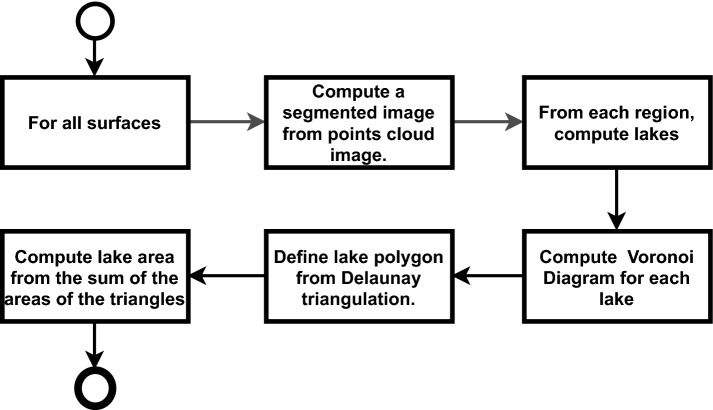
General process for computing valley areas.

In order to classify the areas, it is necessary to carry out some steps of information preparation, up to the computation of the valley areas defined by lakes. Figure 4 presents the general process of the algorithm for calculating areas associated with roughness.

The general process for computing valley areas for each surface is defined as:

1. The first step is to segment the sample into regions whose points are below the adjustment level. This step is detailed in the “Segmentation and classification step of valley regions” section.
2. From the points defined as valley points, identified with black pixels in the segmented surface image, all “lakes”, regions of connected points (neighbors) and that have an area, are computed. The section “Computation step of lake regions of valleys” describes the computation that defines lakes and other information that is extracted from them.
3. For each lake, from its edge points, the Voronoi diagram is computed, and triangulation performed by the Delaunay method. In this way, it is possible to assemble the polygon that surrounds the lake area.
4. From the triangles that form the polygon that surrounds the lake, the area of the lake is computed, adding the area of the triangles. The sum of surface lake areas ($\triangle {_S}$), is the basis for calculating the surface valley area index ($\triangle {_T}$) and average valley area ($\ triangle{_{avg}}$). For purposes of comparison and evaluation of the areas, the standard deviation of the valley areas ($\triangle {_{sdv}}$), smaller area ($\triangle {_{min}}$) and larger area ( $\triangle {_{max}}$).

### Segmentation and classification step of valley regions

To segment and classify lake regions, the point cloud is represented in a 2D image as a height map. From it, a binary image is created for separating pixels into a valley or peak, which is used to create an image that represents the segments of the sample’s valley regions.

**Figure 5:**
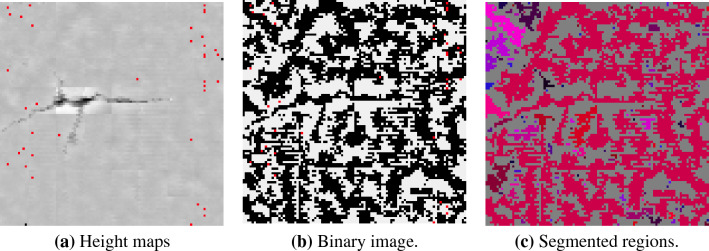
Process of segmentation and classification of valley regions.

In the height map, each point on the cloud is associated with a pixel in the image. Depending on the absolute distance (height) of the point from the adjustment level and the minimum and maximum peak valley values, the corresponding pixel receives an associated gray tone, respectively, on the scale from black to white [0..1]. If the point is below the adjustment level, the corresponding pixel is grayed out from medium gray (0.5) to black (0.0); if a point is above the adjustment level, the pixel is grayed out from medium gray (0.5) to white (1.0). In the image of the height map, it is possible to perceive topography information, including parts of the surface that have a level of waviness information or even relief (as shown in Fig. 5a).

From the height map image, a binary image is created (Fig. 5b), separating the valley points from the peak points. This binarization process is a pre-processing for the segmentation and classification of the valley areas (Fig. 5c). For each pixel of the height map image, one pixel of the binary image is created. The color of this pixel will be white (1.0) if the map pixel is greater than medium gray (0.5); or it will be black if the map pixel is less than or equal to (0.5). The binary image contains only the pixel separation above or below the adjustment level. Regions need to be separated into different sets or segments.

From the binary image, the process of segmentation and classification of the pixels of connected valley areas is carried out, in order to identify and separate the valley regions. A valley region is a collection of connected black pixels. To define a region, the binary image is traversed and, for the first black pixel that was not visited (not sorted by the algorithm), this pixel is assigned to a valley region object and its nearest neighboring pixels (left, top, right and bottom) are also visited, sorted and stacked for verification. These neighboring pixels also go through the same process. This process is repeated until all connected points have been visited and classified, thus forming a valley region. Then the algorithm identifies the next unvisited black pixel not connected to any region and performs the same process recursively. And so on until all points in the binary image are visited and classified. Figure 5c shows the result of the segmentation and classification process, where each region of segmented valley pixels has a different color.

### Computation step of lake regions of valleys

The valley lake region computation step is performed after the sample valley region segmentation step. The input information is the image of segmented regions and, for each surface region, the lakes in the region are identified. All pixels of each lake in a region are grouped in a list and the respective points of the cloud are grouped in another list, to execute the geometric modeling process.

**Figure 6:**
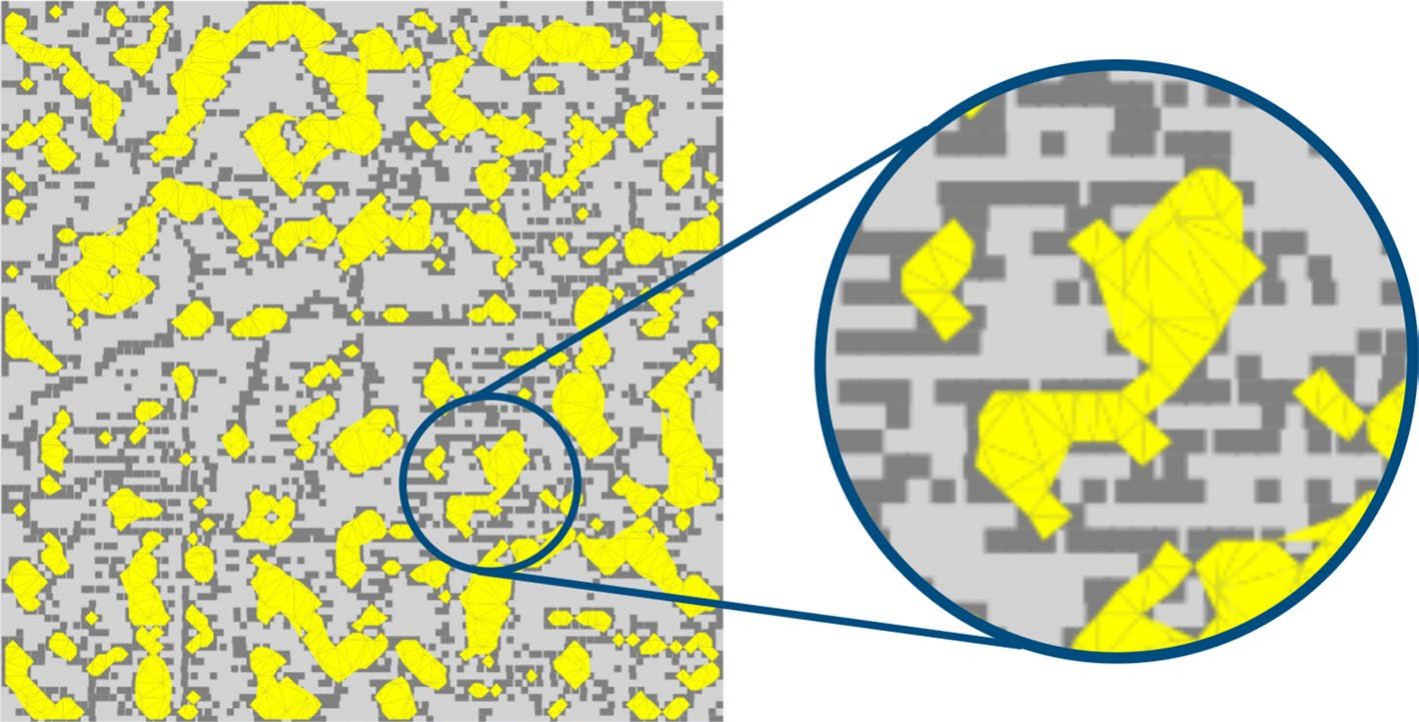
Classification of valley regions in lakes.

After detecting all lakes in all regions, geometric modeling of each lake is performed. The objective is to create a polygonal representation for each lake, to calculate its area and, consequently, calculate the total lake area of a surface. The total surface lake area value is used to determine the valley area index ($\triangle {_T}$) and average valley area ($\triangle {_{avg}}$ parameters, representing related parameters the contact area. Figure 6 is the representation of lakes on a surface. Featured, an example of geometry associated with a lake.

The lake’s geometry is constructed from the organization of the lake’s edge points. Figure 6 shows the complex nature of the shape of the valley regions in the sampled surfaces. They are concave and complex shapes. The Voronoi diagram makes it easy to identify the connections of a point with its neighbors, so the first step is to create a Voronoi diagram from the lake’s edge points, those that define the shape’s perimeter.

After constructing the Voronoi Diagram, the Delaunay^41^ triangulation is performed, connecting reference points of neighboring cells in the diagram. Therefore, the geometry of a lake is defined by the mesh of triangles.

In addition to the polygonal representation of the lake, triangles are also used to calculate the area of valleys. Therefore, let $\triangle$ be the function for calculating the area of a triangle and $\triangle {_L}$ the area of a lake, defined as the sum of the areas of the *T* triangles that make up its geometry:

$$\begin{aligned} \triangle {_L} = \displaystyle \sum _{i=1}^{T} \triangle {i} \end{aligned}$$

And the total surface lake area, which defines the base value for the surface valley area index ($\triangle {_S}$), is calculated by summing the areas of all the $\triangle {_L}$ of *L* surface lakes:

$$\begin{aligned} \triangle {_S} = \displaystyle \sum _{i=1}^{L} \triangle {_L}i \end{aligned}$$

The value $\triangle {_S}$ cannot be used directly, as the total surface area may vary due to the readability of the equipment. The equipment can exceed the limit defined in the parameterization of the reading process. Furthermore, it is important to ensure a basis for comparison between surfaces of any dimension. To ensure this comparison on the same basis, and finally to define the valley area index parameter ($\triangle {_T}$), we normalize the value $\triangle {_S}$, dividing $\triangle {_S }$ by the total surface area ($\square {_{Surface}}$).

The calculation of $\square {_{Surface}}$ is given by:

$$\begin{aligned} \square {_{Surface}} = (x_1 - x_0) \times (y_1 - y_0) \end{aligned}$$

To calculate $$\triangle {_T}$$:

$$\begin{aligned} \triangle {_T} = \frac{\triangle {_S}}{\square {_{Surface}}} \end{aligned}$$

To calculate $\triangle {_{avg}}$ parameter:

$$\begin{aligned} \triangle {_{avg}} = \frac{\triangle {_S}}{L} \end{aligned}$$

Finally, the histogram of the areas is computed, where it is possible to verify the distribution of the areas, in a relation of area size versus frequency along the surface. For this histogram, 20 ranges of area representation were determined, starting from the smallest area among all the $\triangle {_{refmin}}$ samples to the largest area among all the $\triangle {_{refmax}}$ samples, defined previously over all $\triangle {_{min}}$ and $\triangle {_{max}}$ values of all surfaces.

**Figure 7:**
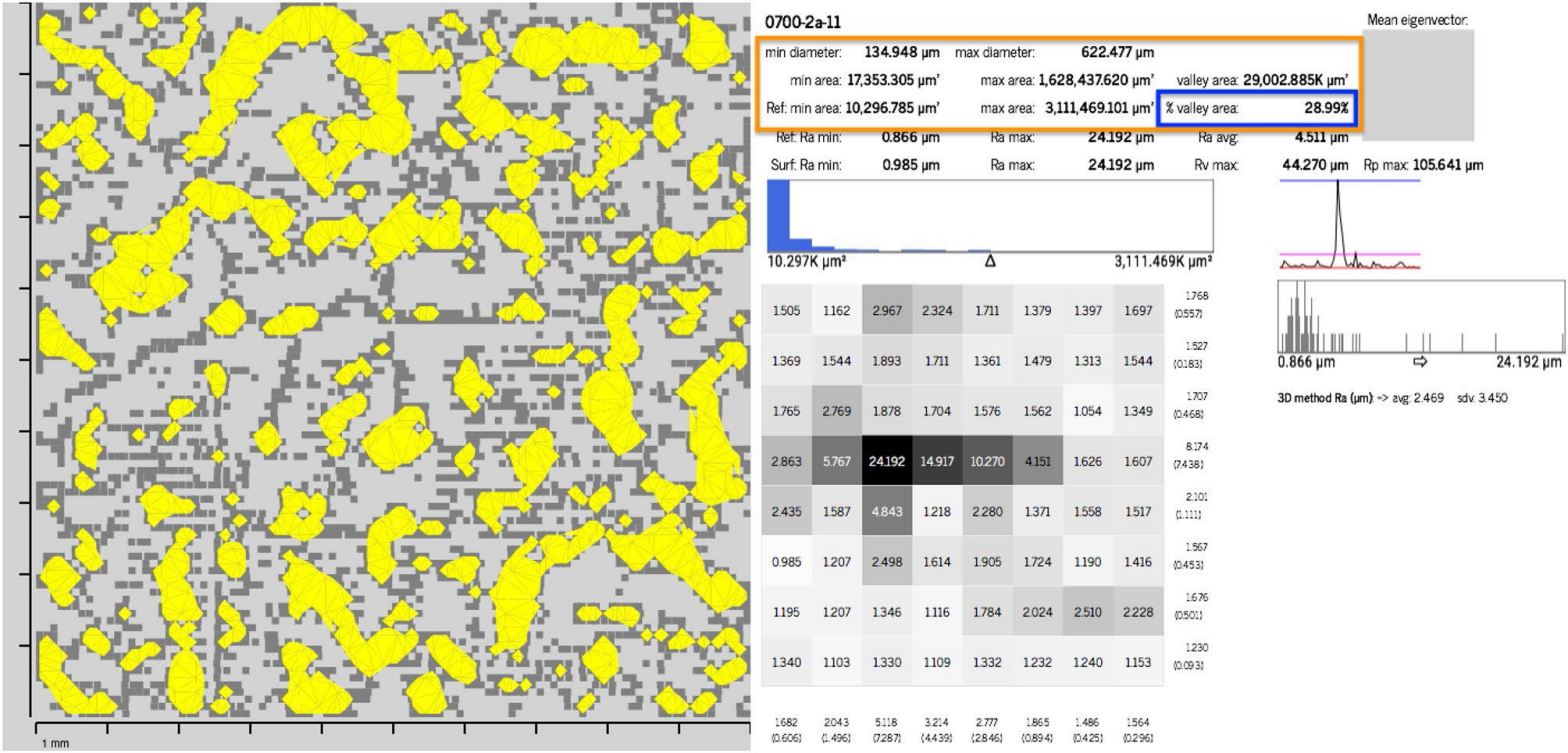
Results generated from the proposed methods.

Figure 7 is an example of all information computed for a sample, containing results developed from the^21^ method and also the adaptations and implementations of the method proposed in this work for roughness determination and evaluation, therefore, correlate with adhesion area determination parameters. In the left half, visual information about the area of the sample valley lakes (yellow pixels) is displayed. In the right half, roughness measurements ($R_a$) of the sample and reference ($R_amin$, $R_amax$ and $R_aavg$) are displayed. In addition to the sample area measurements ($\triangle {_S}$ and $\triangle {_T}$) and reference ($\triangle {_{min}}$ and $\triangle {_{max}}$) . The graphical information presented in^21^ (roughness signature, $R_a$ behavior graphs and $R_a$ value distribution) are shown in the lower right corner. The parameters related to the valley area are highlighted in orange and the valley area index ($\triangle {_T}$) of the sample is highlighted in blue.

## Method validation

To validate the method and demonstrate its operation, some tests were carried out on 500 samples of red clay ceramic substrate. The samples come from 5 different brickyards and, for each brickyard, 10 blocks were acquired, prepared and read with 3D laser equipment (LiDAR). The purpose of making this sample selection is to provide variability to the test in relation to the manufacturing process of ceramic blocks, since the blocks of each brickyard have different mineral composition, mixing and conformation process and fire temperature. All samples were read according to the procedure described in the reference work^21^. To read 3D data, the Starrett AV300+ equipment was used with an X and Y resolution of $E2 = 1.9~\mu m + 5L/1000$ and a Z resolution of $E1= 2.5~\mu m + 5L/1000$, scale resolution of $0.1~\mu m$.

The purpose of the test is to verify if the method is effective for roughness computation and also to demonstrate how to use the valley area index ($\triangle {_T}$) and average valley area ($\triangle {_{avg) parameters }}$). To test the effectiveness of the method, the roughness parameters are also computed, as is done in^21^.

Regarding the parameters referring to the valley areas, the hypothesis is that valley areas with larger size on average favor a larger contact area between the ceramic substrate and the cement matrix, allowing a larger adhesion area, and in larger valleys there is greater probability of contact with the base.

To validate the proposed method, 5 analyzes were performed:

1. Direct comparative analysis using the parameters valley area index ($\triangle {_T}$) and average valley area ($\triangle {_{avg}}$), to determine which brickyard presents blocks with greater adherence favoring.
2. Hypothesis testing, but specifically the Z-test, on the valley mean area data ($\triangle {_{avg}}$), at a significance level of $5\%$, to verify the area data valley averages are significantly higher from one brickyard to another. In this way, it is possible to statistically indicate which would be the brickyards with greater or lesser average valley area ($\triangle {_{avg}}$) and, consequently, those that produce blocks that favor the adhesion area, with greater penetration of particles of the cement matrix.
3. Comparative analysis of $\triangle {_T}$ to verify the distribution of values of $\triangle {_T}$ by the blocks of each brickyard and to indicate which brickyards favor the adhesion area.
4. Validation of the multiple plane method proposed in this work to compute surface roughness. The objective of this test is to compare values of $R_a$ calculated for the blocks of each brickyard and indicate the brickyard that has the greatest roughness and that, theoretically, favors the bonding area.

### Analysis of parameters related to valley area

**Figure 8:**
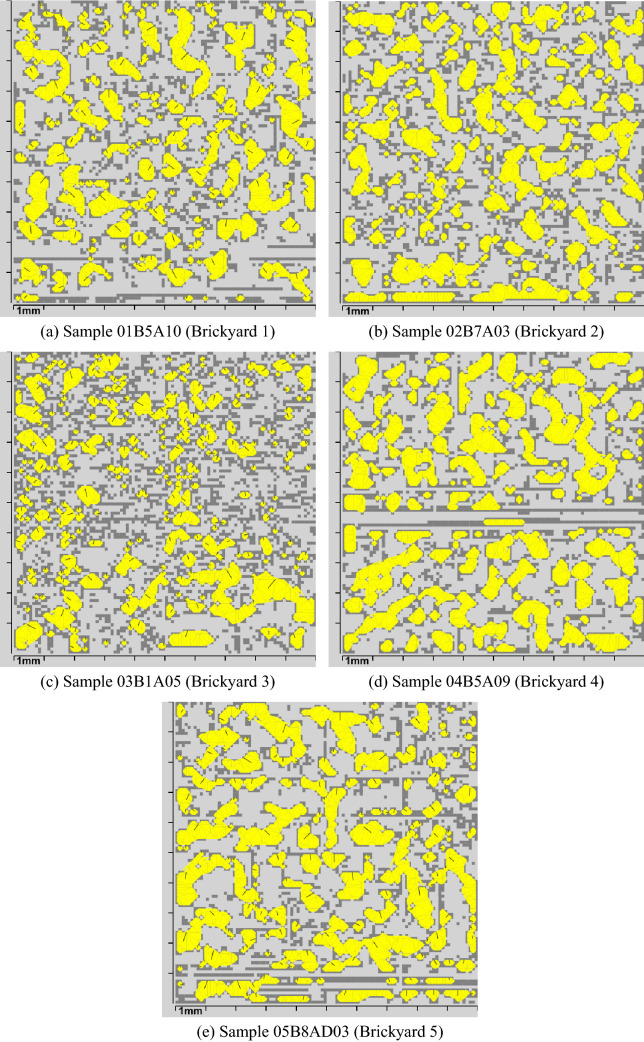
Results that demonstrate the calculation of areas. In (**a**) a result of brickyard 1, in (**b**) of brickyard 2, in (**c**) of brickyard 3, in (**d**) of brickyard 4 and in (**e**) of brickyard 5.

To demonstrate the use of the parameters valley area index ($\triangle {_T}$) and the average valley area ($\triangle {_{avg}}$), some results presented in Table 1 and Fig. 8 and statistical analysis results are also presented.

**Table 1:** Values $\triangle {_{avg}}$, $\triangle {_{T}}$ and $\triangle {_{T}avg}$ from each of the five brickyards shown in the Fig. 8.

Brickyard	Sample	Sample $\triangle {_{avg}}$	Sample $\triangle {_{T}}$	Brickyard $\triangle {_{T}avg}$
Brickyard 1	01B5A10	$0.1509\,{\text{ mm}}^2$	$27.62\%$	$27.60\%$
Brickyard 2	02B7A03	$0.1272\,{\text{ mm}}^2$	$30.02\%$	$30.01\%$
Brickyard 3	03B1A05	$0.0799\,{\text{ mm}}^2$	$22.85\%$	$22.87\%$
Brickyard 4	04B5A09	$0.2129\,{\text{ mm}}^2$	$33.43\%$	$33.43\%$
Brickyard 5	05B8AD03	$0.2040\,{\text{ mm}}^2$	$34.06\%$	$34.00\%$

In this analysis, 5 samples considered representative were selected, one for each brickyard. Of all the samples from the same brickyard, the most representative sample was considered the one with a value of $\triangle {_T}$ closest to the average value of $\triangle {_{T}}$ of the respective brickyard ($\triangle { _{T}avg}$).

In Fig. 8, the yellow pixels represent the valley areas of each sample. Visually, it is not possible to accurately assess the results, but it is noticeable that some surfaces have larger valley areas than others, while others have more valley areas, but with smaller sizes. This impacts the parameters related to valley areas. Those surfaces with larger yellow areas, as can be seen in Fig. 8 for brickyards 4 and 5, have a higher $\triangle {_{avg}}$ value than other surfaces, such as brickyards 1, 2 and 3 in the same figure. The difference between the roughness can be analyzed numerically, observing the values of Table 1. Brickyard 4 and 5 have the highest values of $\triangle {_{avg}}$ and ($\triangle {_{T}}$), being considered the one with the most favorable roughness profile for adhesion.

#### Area parameter considerations

Regarding the parameters $\triangle {_T}$ and $\triangle {_{avg}}$, the joint analysis of the parameters is proposed, since the size and shape of the concentration areas of particles in the cement matrix varies between the samples.

It is estimated that the average valley area parameter ($\triangle {_{avg}}$) may indicate better adherence than the valley area index parameter ($\triangle {_T}$), as it has a greater relationship with the particle diameter of the cementitious matrix. If two samples *A* and *B* have values of $\triangle {_{avg}}$ very close, the one with the highest value $\triangle {_T}$ indicates a greater favoring of the adhesion area.

### Verification of average area of valleys

In this evaluation, the values of the average valley area ($\triangle {_{avg}}$) are used. The objective is to demonstrate that the average valley areas of one brickyard, at a significance level of $5\%$, are significantly larger than the average areas of the others.

The test result allows us to observe which brickyard produces the blocks with the highest average valley area index ($\triangle {_{avg}}$) and, consequently, blocks that theoretically favor the adhesion area. The tables 2 and 3 present the values of ($\triangle {_{avg}}$) computed for the test, separated by brickyard, by block and by sample.

**Table 2:** Average valley area values ($\triangle {_{avg}}$), computed for blocks 1 to 5 of the 5 brickyards in the test. Values are presented in $mm^2$.

Surface	Brickyard 1	Brickyard 2	Brickyard 3	Brickyard 4	Brickyard 5
B1A01	0.1513	0.1258	0.0846	0.1961	0.1912
B1A02	0.1441	0.1496	0.1066	0.1808	0.2069
B1A03	0.1519	0.1281	0.0702	0.1911	0.2043
B1A04	0.1272	0.1406	0.0680	0.1763	0.2206
B1A05	0.1440	0.1245	0.0799	0.1636	0.2251
B1A06	0.1327	0.1583	0.0866	0.1821	0.2400
B1A07	0.1372	0.1254	0.0735	0.1656	0.1822
B1A08	0.1058	0.1361	0.1049	0.1682	0.2506
B1A09	0.1208	0.1341	0.0896	0.1878	0.2534
B1A10	0.1312	0.1764	0.0864	0.1916	0.2825
B2A01	0.1029	0.1598	0.1227	0.2075	0.1919
B2A02	0.1178	0.1489	0.0765	0.2008	0.1798
B2A03	0.1073	0.1531	0.1319	0.1793	0.2272
B2A04	0.1271	0.1489	0.1134	0.1758	0.1901
B2A05	0.1201	0.1323	0.0990	0.2072	0.1459
B2A06	0.1206	0.1277	0.0906	0.1603	0.1390
B2A07	0.1158	0.1490	0.0839	0.1906	0.2168
B2A08	0.1306	0.1688	0.0763	0.1642	0.1926
B2A09	0.1232	0.1391	0.0640	0.1922	0.2083
B2A10	0.1331	0.1521	0.0906	0.1379	0.2370
B3A01	0.1336	0.1475	0.2252	0.2048	0.2078
B3A02	0.1117	0.1561	0.0874	0.1939	0.1984
B3A03	0.1118	0.1515	0.1109	0.1622	0.2568
B3A04	0.1166	0.1394	0.1028	0.1904	0.1751
B3A05	0.1175	0.1210	0.1018	0.1710	0.1788
B3A06	0.1388	0.1348	0.1068	0.1950	0.2331
B3A07	0.1248	0.1497	0.1288	0.1673	0.1891
B3A08	0.1218	0.1885	0.1703	0.1576	0.2476
B3A09	0.1386	0.1583	0.0910	0.1497	0.2104
B3A10	0.1174	0.1564	0.0905	0.1854	0.2442
B4A01	0.1239	0.1684	0.1848	0.1978	0.2008
B4A02	0.1555	0.1628	0.0872	0.1945	0.2654
B4A03	0.1177	0.1654	0.0816	0.2010	0.2074
B4A04	0.1147	0.1415	0.0802	0.1644	0.2145
B4A05	0.1413	0.1461	0.1348	0.1869	0.2586
B4A06	0.1265	0.1302	0.1179	0.1496	0.2414
B4A07	0.1259	0.1502	0.0887	0.1920	0.2785
B4A08	0.1309	0.1670	0.0734	0.1917	0.2499
B4A09	0.1075	0.1543	0.1321	0.1869	0.1833
B4A10	0.1014	0.1424	0.0822	0.2137	0.2233
B5A01	0.1068	0.1614	0.1524	0.1760	0.1887
B5A02	0.1176	0.1766	0.1016	0.2353	0.2083
B5A03	0.1133	0.1335	0.0881	0.2068	0.2015
B5A04	0.1317	0.1749	0.0669	0.1519	0.1932
B5A05	0.1053	0.1633	0.1069	0.1848	0.2228
B5A06	0.1236	0.1706	0.1804	0.2379	0.2404
B5A07	0.1406	0.1747	0.0865	0.1914	0.1787
B5A08	0.1384	0.1571	0.0962	0.2045	0.1984
B5A09	0.0980	0.1584	0.0795	0.2129	0.1820
B5A10	0.1509	0.1646	0.1356	0.1908	0.2100

**Table 3:** Average valley area values ($\triangle {_{avg}}$), computed for blocks 6 to 10 of the 5 brickyards in the test. Values are presented in $\text{mm}^2$.

Surface	Brickyard 1	Brickyard 2	Brickyard 3	Brickyard 4	Brickyard 5
B6A01	0.1358	0.1674	0.0830	0.2039	0.2162
B6A02	0.1027	0.1140	0.0904	0.2157	0.2169
B6A03	0.1093	0.1478	0.0790	0.1833	0.2432
B6A04	0.0938	0.1626	0.1001	0.1734	0.2559
B6A05	0.1103	0.1399	0.0940	0.1611	0.2458
B6A06	0.1132	0.1551	0.0950	0.2110	0.2590
B6A07	0.1131	0.1525	0.0972	0.1697	0.2823
B6A08	0.1198	0.1511	0.1079	0.1946	0.2415
B6A09	0.1014	0.1485	0.0853	0.1630	0.2353
B6A10	0.1282	0.1561	0.0728	0.1762	0.2612
B7A01	0.1296	0.1361	0.1020	0.1690	0.2045
B7A02	0.1129	0.1513	0.0988	0.1656	0.2795
B7A03	0.1103	0.1272	0.1207	0.2061	0.2190
B7A04	0.1223	0.1559	0.0818	0.1876	0.2111
B7A05	0.1343	0.1282	0.1060	0.2016	0.2225
B7A06	0.1167	0.1686	0.0832	0.1838	0.1968
B7A07	0.1377	0.1461	0.0881	0.2081	0.2346
B7A08	0.1453	0.1118	0.1014	0.2000	0.2457
B7A09	0.1317	0.1623	0.0727	0.1622	0.1930
B7A10	0.1457	0.1674	0.0791	0.1754	0.2370
B8A01	0.1258	0.1342	0.0892	0.1683	0.1977
B8A02	0.1375	0.1634	0.1164	0.1840	0.1804
B8A03	0.1312	0.1377	0.1032	0.1648	0.2040
B8A04	0.1297	0.1244	0.1202	0.1409	0.2196
B8A05	0.1428	0.1336	0.0856	0.1816	0.2163
B8A06	0.1056	0.1255	0.1201	0.1791	0.2252
B8A07	0.1202	0.1299	0.1399	0.1944	0.1822
B8A08	0.1131	0.1554	0.1120	0.1768	0.3125
B8A09	0.1006	0.1316	0.1042	0.1996	0.2011
B8A10	0.1230	0.1350	0.0987	0.2098	0.1961
B9A01	0.0986	0.1344	0.0820	0.2055	0.2202
B9A02	0.1214	0.1663	0.1096	0.1812	0.1666
B9A03	0.1403	0.1680	0.0839	0.2117	0.2347
B9A04	0.1061	0.1538	0.0981	0.1754	0.2908
B9A05	0.1246	0.1714	0.0975	0.1980	0.2451
B9A06	0.1196	0.1891	0.1172	0.2263	0.2094
B9A07	0.1361	0.1238	0.1163	0.1629	0.1992
B9A08	0.1123	0.1809	0.1344	0.1678	0.2311
B9A09	0.1130	0.1647	0.1213	0.2036	0.1968
B9A10	0.0916	0.1929	0.1255	0.1642	0.2655
B10A01	0.1131	0.1516	0.1028	0.1675	0.2515
B10A02	0.1334	0.1385	0.1058	0.1546	0.2488
B10A03	0.1443	0.1284	0.0900	0.2163	0.2214
B10A04	0.1189	0.1293	0.0852	0.2063	0.2301
B10A05	0.1301	0.1135	0.0930	0.2439	0.2175
B10A06	0.1312	0.1119	0.0998	0.1910	0.2317
B10A07	0.1385	0.1299	0.1128	0.1740	0.2496
B10A08	0.1545	0.1303	0.0829	0.2276	0.2401
B10A09	0.1032	0.1511	0.0862	0.2024	0.2074
B10A10	0.1335	0.1224	0.0918	0.1762	0.2975

Table 4 presents the result of testing one area versus the others to determine whether it is possible to assert, at a significance level of $5\%$, that the average valley area index data ( $\triangle {_{avg}}$) of one brickyard is significantly larger than that of other brickyards. In this hypothesis test, it is important that the sample with the greatest variance is the first variable, so only one comparison result between two brickyards was placed in the table. When the value of *Z* is greater than the value $Z_{critical} = 1.959963985$, the value $\triangle {_{avg}}$ is considered to be significantly greater than the $\triangle {_{avg}}$ value of another brickyard.

**Table 4:** Z-test result for area average ($\triangle {_{avg}}$) data.

	Brickyard 1	Brickyard 2	Brickyard 3	Brickyard 4	Brickyard 5
Brickyard 1			7.469695041		
Brickyard 2	10.55273669		14.79163222		
Brickyard 3					
Brickyard 4	24.47185306	13.69900065	25.43899564		
Brickyard 5	27.86465433	19.94408906	29.20062951	22.15775325	

Considering the values of Table 4, it is possible to state that the values of Brickyard 5 are significantly higher than the average area values ($\triangle {_{avg}}$) of other brickyards. Considering exclusively the Z-test analysis and the hypothesis that the greater the contact area, the better the adhesion, it is estimated that the blocks from brickyard 5 favor a greater adhesion area than the blocks from the other studied brickyards.

### Comparison of valley area indexes

**Figure 9:**
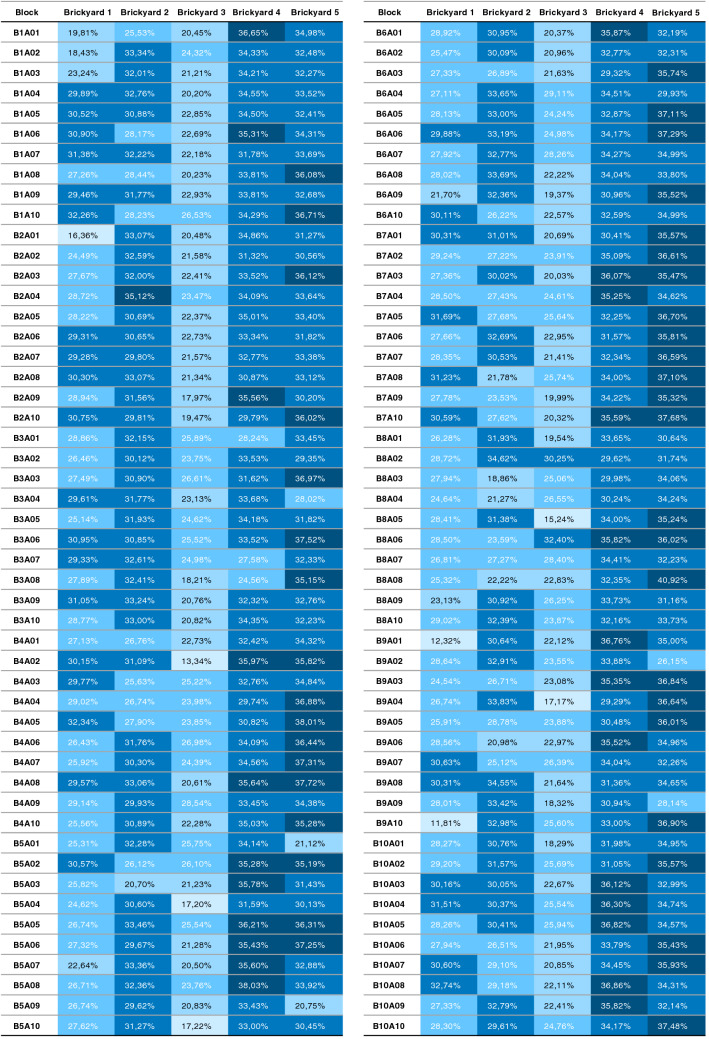
Valley area index ($\triangle {_T}$) of all samples, scaled from smallest (light) to largest (dark) ($\triangle {_T}$).

For this analysis, the values of the valley area index ($\triangle {_T}$) were considered, separated by brickyard, by block and by sample. Table 5 presents the average and standard deviation data on the comparison data of valley area indices ($\triangle {_T}$) presented in Fig. 9.

**Table 5:** Average and standard deviation of the valley area indices ($\triangle {_T}$).

	Brickyard 1	Brickyard 2	Brickyard 3	Brickyard 4	Brickyard 5
Average	27.60	30.01	22.87	33.43	34.00
Standard deviation	3.56	3.39	3.13	2.28	3.11

According to the information in Table 5, brickyards 4 and 5 present an average of $\triangle {_T}$ significantly higher than the average of $\triangle {_T}$ of the other brickyards. Also considering the standard deviation data, it is verified that the values $\triangle {_T}$ of these two brickyards are similar.

Another way of evaluating the $\triangle {_T}$ parameter in relation to the brickyard samples is the separation into value ranges. Like a histogram, the objective is to visualize the distribution of values in order of relevance (higher value, more relevant). To do so, the minimum ($\triangle {_{min}}$) and maximum ($\triangle {_{max}}$) values of all samples (Figure values) are determined in FIg. 9). The minimum ($\triangle {_{min}}$) and maximum ($\triangle {_{max}}$) values define the histogram range of $\triangle {_T}$ and, within that range, five evenly distributed ranges are defined (shown in Table 6).

**Table 6:** Ranges for grouping values of $\triangle {_T}$.

Range	Lowest percentage	Highest percentage
Range 1	11.81	17.63
Range 2	17.63	23.46
Range 3	23.46	29.28
Range 4	29.28	35.10
Range 5	35.10	40.92

From the values $\triangle {_T}$ (shown in Fig. 9) the histogram is composed, counting the value $\triangle {_T}$ of each sample in the respective representation range. Table 7 shows the amount of $\triangle {_T}$ per representation band. The lines are in the order of the brickyard with the lowest average $\triangle {_T}$ to the brickyard with the highest average $\triangle {_T}$.

**Table 7:**
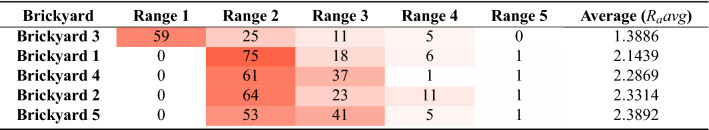
Amount of values of $\triangle {_T}$ per brickyard and per representation range. Rows are ordered by the average $\triangle {_{T}}$ of the brickyard.

The results indicate the greatest distribution of areas with the greatest $\triangle {_T}$ for the 4 and 5 brickyard, as they concentrate most of the values in the 4 and 5 ranges. Brickyard 3, on the other hand, has the smallest number of areas with the highest range of representation (ranges 4 and 5) and concentrates a greater amount of $\triangle {_T}$ values in the representation ranges 2 and 3. Furthermore, as shown in the comparison of Table 5, brickyard 3 has in average smaller values of $\triangle {_T}$. In the same table, it can be seen that brickyards 4 and 5 have, on average, the highest values of $\triangle {_T}$.

**Figure 10:**
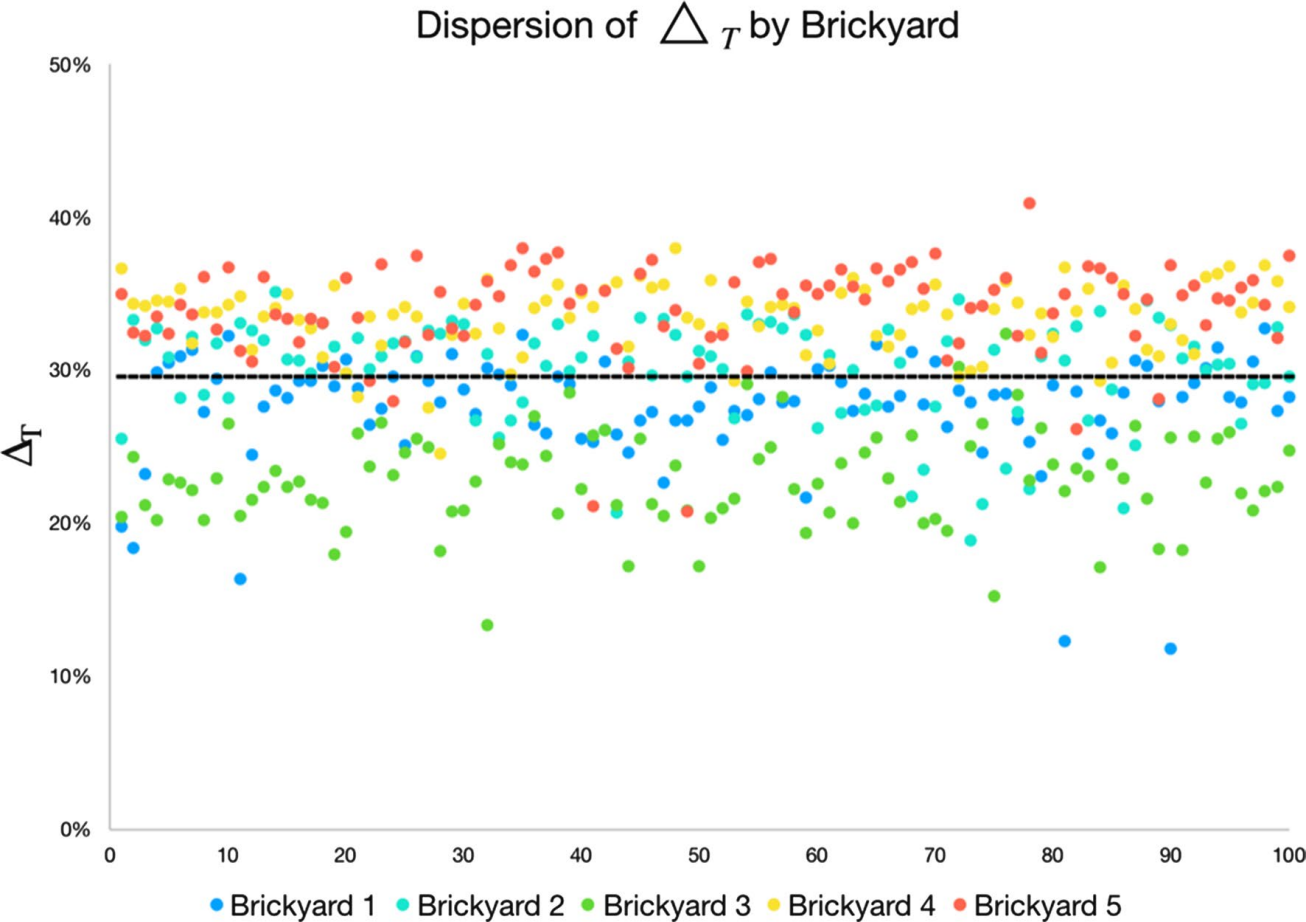
Scattering of $\triangle {_T}$ by brickyard. In black the average line of $\triangle {_T}$ of the brickyards.

Figure 10 presents an overview of the average of all $\triangle {_T}$ values of the blocks of each brickyard in a scatter plot. As can be seen in the graph, brickyard 3 (dots in green) is mostly below average, indicating by the analysis of $\triangle {_T}$ that this brickyard would have less favor in the adhesion area than the others.

According to what was presented in Figs. 9 and 10 and in Tables 5, 7, brickyard 5 has the highest frequency of higher total area index values. Brickyard 4 presents similar values and with a smaller standard deviation, indicating that it also has a good distribution and standardization of contact area indices $\triangle {_T}$. In this analysis and according to the hypothesis that the greater the valley area index ($\triangle {_T}$), the better the adherence, it is possible to state that brickyards 4 and 5 have better $\triangle {_T}$ and tend to favor the adhesion area. Consequently, as brickyard 3 has a lower frequency of $\triangle {_T}$ values in the higher representation ranges, it is concluded, by the same criterion, that the blocks produced by it do not favor adhesion as much as the other brickyards, when compares the way in which the grains of the same paste or mortar composed of fine grains on a micrometric scale are accommodated on the rough surface of the block.

### Average roughness comparison

**Figure 11:**
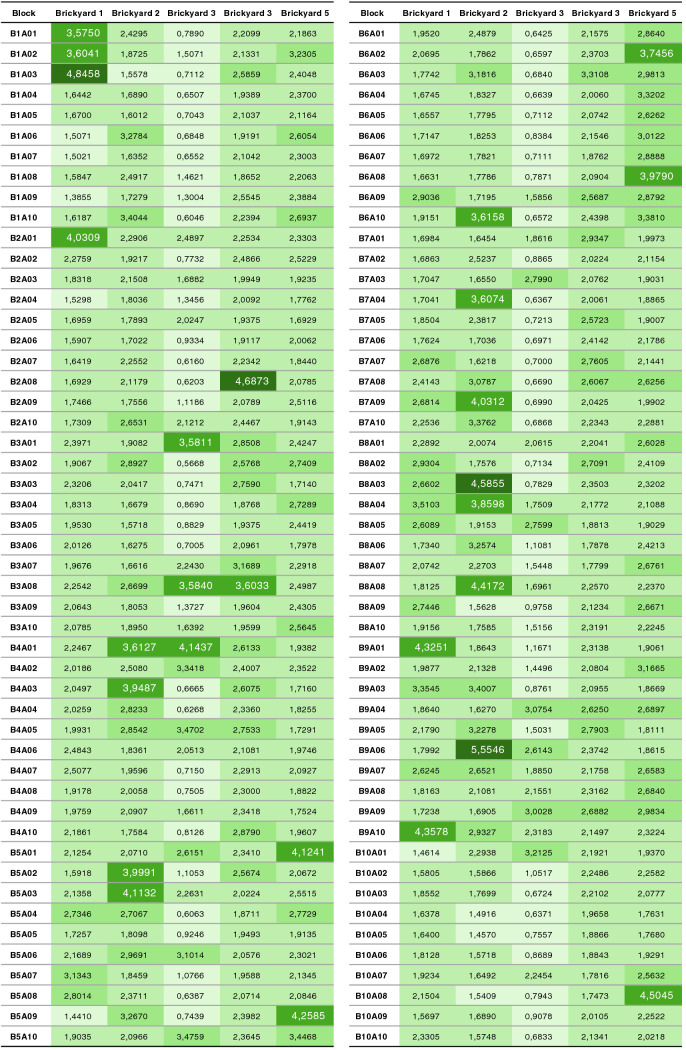
Average roughness ($R_aavg$) of all samples, scaled from smallest (light) to largest (dark) ($R_aavg$).

In this test step, the average roughness data $R_aavg$ for all blocks of all brickyards are computed. The objective is to compare the average roughness data between the brickyards to determine which of them favors adhesion with greater intensity. The hypothesis used in this test is that the brickyard that presents the highest value $R_aavg$ favors more adherence. The data for $R_aavg$ are shown in Fig. 11.

As was done in the area analysis, the data of $R_aavg$ between brickyards is also compared. Table 8 presents the average and standard deviation data of $R_aavg$ on the data presented in Fig. 11.

**Table 8:** Average and standard deviation of the average roughness ($R_aavg$) of the samples.

	Brickyard 1	Brickyard 2	Brickyard 3	Brickyard 4	Brickyard 5
Average	2.1439	2.3314	1.3886	2.2869	2.3892
Standard deviation	0.6656	0.8339	0.8991	0.4195	0.5746

Like a histogram the data from $R_aavg$ is grouped and counted into value ranges. The objective is to visualize the distribution of values in order of relevance (highest value, most relevant). Therefore, the minimum ($R_amin$) and maximum ($R_amax$) values of $R_aavg$ of all samples are determined (values in Fig. 11). The minimum ($R_amin$) and maximum ($R_amax$) values define the histogram range of $R_aavg$ and, within this range, five uniformly distributed ranges are defined (presented in Table 9) .

**Table 9:** Ranges for grouping values of $R_aavg$.

Range	Lowest $R_aavg$	Highest $R_aavg$
Faixa 1	0.5668	1.5644
Faixa 2	1.5644	2.5619
Faixa 3	2.5619	3.5595
Faixa 4	3.5595	4.5570
Faixa 5	4.5570	5.5546

From the values $R_aavg$ (shown in Fig. 11) the histogram is composed, counting the value $R_aavg$ of each sample in the respective representation range. Table 10 shows the amount of $R_aavg$ per representation range. The lines are fitted from the brickyard with the lowest average $R_aavg$ to the brickyard with the highest average $R_aavg$.

**Table 10:**
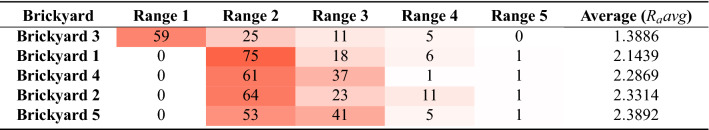
Ranges for grouping values of $R_aavg$. Rows are sorted by average ($R_aavg$).

The results indicate the greatest distribution of areas with the highest $R_aavg$ for the brickyards 2, 4 and 5, as they concentrate most of the values of $R_aavg$ in the ranges 3 and 4. The brickyard 3, on the other hand, has the smallest number of areas with the highest representation range (lanes 3, 4 and 5) and concentrates the greatest amount of values $R_aavg$ in the representation range 1. Furthermore, as shown in the comparison of Table 8, brickyard 3 has average values of $R_aavg$ smaller. In the same table, it can be seen that brickyards 2, 4 and 5 have, on average, the highest values of $R_aavg$.

**Figure 12:**
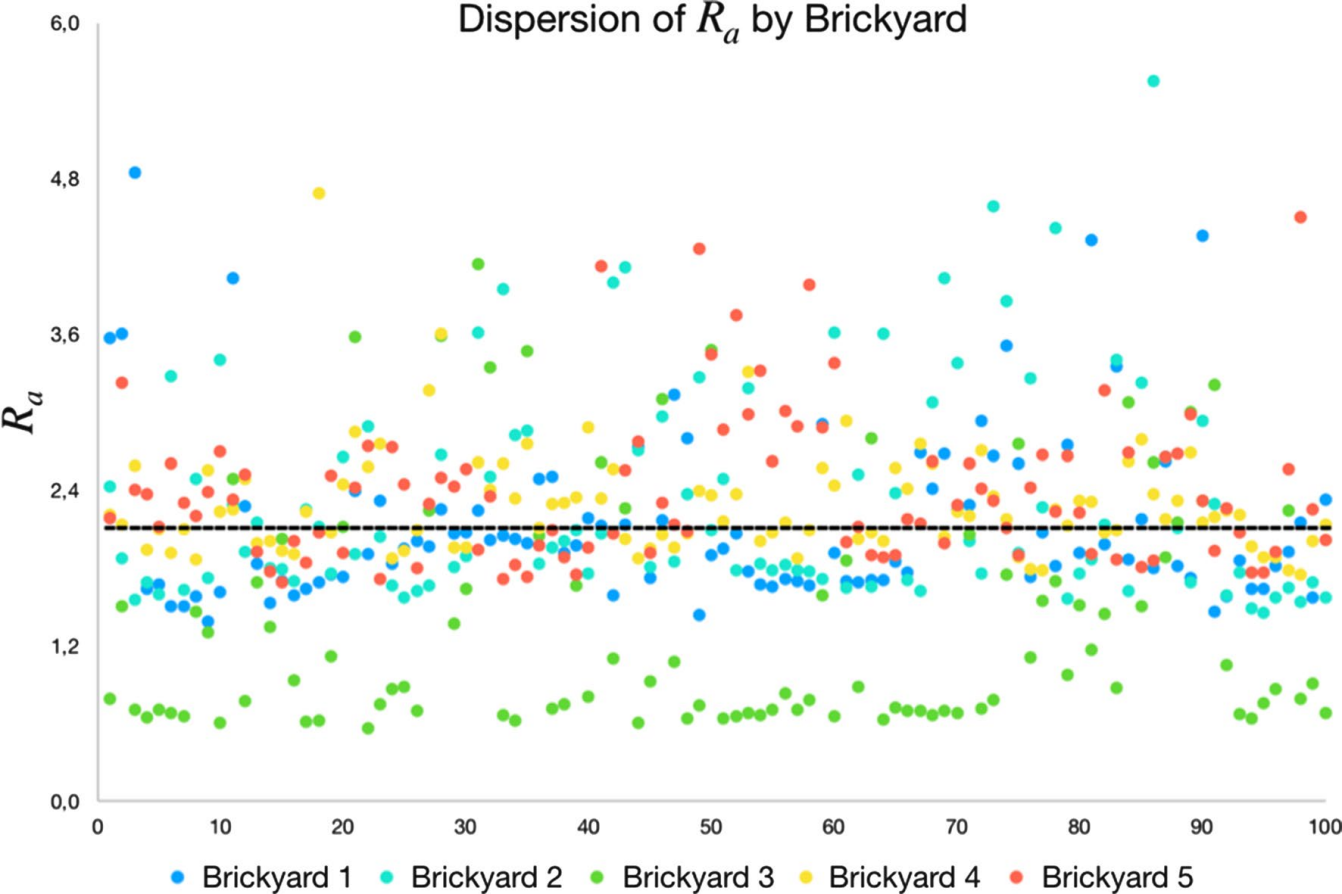
Average roughness distribution by brickyard. In black the $R_aavg$ average line of all brickyards.

Figure 12 presents an overview of all $R_aavg$ values per block for each brickyard in a scatter plot. As can be seen in the graph, the values of $R_aavg$ of the blocks of brickyard 3 (dots in green) are mostly below the average, indicating by the analysis of $R_aavg$ that this brickyard would have less adherence than the others.

According to what was presented in the Figs. 11 and 12 and in Tables 8 and 10, brickyard 5 and 2 have the highest frequency of higher average roughness values. Brickyard 4 presents similar values and with a smaller standard deviation, indicating that it also has a good distribution and standardization of average roughness. In this analysis and according to the hypothesis that the higher the average roughness, the more favorable to adherence, it is possible to affirm that brickyards 2, 4 and 5 have greater $R_aavg$ and favor adherence. Consequently, as brickyard 3 has a lower frequency of values $R_aavg$ in the ranges with greater representation, it can be concluded, by the same criterion, that the blocks produced by it do not favor as much adherence as the other brickyards.

## Conclusion

Analysis of roughness parameters is recognized^21,23,26,34,37,39,42^ an effective way to characterize surfaces, in relation to the mechanical interlock between the substrate and the cementitious matrix. This work presented an evolution to the method described in^21^ with the computation of surface roughness from a grid of planes (one plane for each region/node of a quadtree tree structure) and from the points that belong to each region (node). The method also presents new parameters for the analysis of adhesion areas, related to the contact surface, as parameters that can be used for quantitative assessment of the adhesion area.

The results demonstrate that the method is effective in determining roughness and that the area-related parameters ($\triangle {_T}$ and $\triangle {_{avg}}$) can be used to compare samples under different production conditions in relation to the area of adhesion between the ceramic substrate and the cementitious matrix.

**Figure 13:**
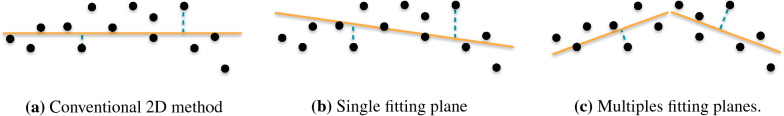
Fitting planes for roughness computation. In (**a**) the conventional 2D method (profile line aligned with *X* axis). In (**b**) the single fitting plane, which considers all points in the point cloud, and in (c) the multiple planes, calculated from the points at each location.

The multiple plane computation method allows a geometric modeling closer to the surface topography. Thus, the roughness values calculated by the method do not consider the surface waviness and better represent the micro-roughness as they are an evolution of the^21^ method, used to calculate $R_a$. The roughness values obtained through the application of this method are smaller compared to^21^, this can be explained by the fact that the planes map surface waviness as the geometry and the points are closer to the respective planes. Figure 13 illustrates the difference between the methods. In (a) an example of conventional 2D methods, which use a single profile line aligned with the *X* axis as a reference. The method proposed in^21^ defines an adjustment plane for the entire point cloud. And in (c) the adjustment planes with points at each location on the surface of the method proposed in this work. At the current stage of the method’s development, there is the possibility that eventually a lack of continuity between the fitting plane of neighbor regions may occur. The real need for changing the method in relation to this behavior is being evaluated and may generate future improvements in the method.

The parameters of valley area index ($\triangle {_T}$) and average valley area ($\triangle {_{avg}}$) proposed to evaluate the surface area of ceramic blocks, with the intention of proposing a coefficient associated with the contact surface, allow the evaluation and comparison of surface samples and indicate which ones favor the largest adhesion area, considering the hypothesis that, the greater the value of the analyzed parameter, the greater the adhesion area provided on the surface. It is estimated that the parameter ($\triangle {_{avg}}$) can better indicate the surface adhesion area, since with this factor it is possible to relate the dimension and the amount of grains of the cement matrix that can fill regions of surface valleys area. However, the parameter $\triangle {_T}$ also provides characteristics of the adhesion area capacity of the ceramic substrate surface. The assumption that was presented in this work is that, if a surface has a value $\triangle {_{avg}}$ significantly higher than the parameters determined on the other surface, then this surface favors the largest adhesion area. However, for surfaces with similar values of $\triangle {_{avg}}$, the one with a higher average value of $\triangle {_T}$ favors more adhesion area.

The developed method can be used for the selection of the type of mortar that will be applied over a given substrate, allowing a better prediction of the adherence of the coating according to the exposure conditions during its lifetime.

It is worth noting that, although the parameters perform a correct mathematical evaluation of the surface area, it is necessary to experiment with physical tests to obtain more accurate diagnoses and validation of these parameters. Besides the relationship with the adherence tests, it is intended the evolution of the method to calculate other parameters related to roughness and contact surfaces, as valleys volume.

## Data Availability

The datasets that were generated and/or analysed during the current study are freely available from the corresponding author on a request.
